# Delay in Seeking Medical Treatment Among Patients With Acute Coronary Syndrome

**DOI:** 10.7759/cureus.17369

**Published:** 2021-08-22

**Authors:** Prashant Panda, Neena Vir Singh, Navjyot Kaur, Prabhjot Kaur, Avneet Kaur, Harleen Kaur Aujla, Khushpreet Kaur, Nishtha Saini, Shakshi Kapoor, Yash Paul Sharma

**Affiliations:** 1 Cardiology, Postgraduate Institute of Medical Education & Research, Chandigarh, IND; 2 Nursing, National Institute of Nursing Education, Postgraduate Institute of Medical Education & Research, Chandigarh, IND; 3 Cardiology, Command Hospital Air Force, Bangalore, IND

**Keywords:** acute coronary syndrome, pharmaco-invasive therapy, pre-hospital delay, revascularization, stemi, thrombolysis

## Abstract

Background

Various Indian registries have documented a delay of more than five hours for acute coronary syndrome patients from onset of symptoms to reaching thrombolysis-enabled centres. We conducted this study to evaluate the factors responsible for pre-hospital delay in acute coronary syndrome patients.

Methods

This was a descriptive cross-sectional study conducted in consecutive acute coronary syndrome patients who reported to the tertiary care medical centre in North India. A standardized tool was used to record the demographic data, socioeconomic status and clinical presentation of patients. All factors which led to pre-hospital delay were noted and the appropriate statistical tests were used for analysis.

Results

A total of 130 patients (males=93, females=37) were included in the study. The median time at which the acute coronary syndrome patients presented to the thrombolysis and percutaneous coronary intervention enabled centre was 490 minutes (range: 20 - 810 minutes) and 710 minutes (range: 45 - 940 minutes) respectively. The various factors responsible for prehospital delay were rural residence (p-value <0.0001), visit to local dispensary (p-value=0.0023), delay in getting transport (p-value=0.03) and misinterpretation of cardiac symptoms (p-value=0.0004). A significant but weak negative correlation was found between per capita income, decision making time and time taken to receive thrombolytic therapy. Out of a total of 83 ST-elevation myocardial infarction patients, only 46 (51.80%) were thrombolysed. Though 69/83 (83.13%) ST-elevation myocardial infarction patients reached thrombolysis enabled centre directly, only nine (10.84%) were thrombolysed at first medical contact; the rest were transferred to the percutaneous coronary intervention-enabled centre without any prior information.

Conclusion

Our study concludes that besides socioeconomic and demographic variables, lack of public awareness, well established public transport & health insurance system lead to significant pre-hospital delays and increase the time to revascularization. Besides, judgemental error on the part of medical practitioners in the peripheries also significantly delays thrombolysis in ST-elevation myocardial infarction patients.

## Introduction

Coronary artery disease (CAD) has reached epidemic status in India [1]. Worldwide, it is currently the most common cause of mortality and morbidity [2-4]. There has been an increase in the prevalence of CAD in India over the last 60 years, from 1% to 9%-10% and <1% to 4%-6% in urban and rural populations respectively [1]. Further, one-fourth of all deaths in India are attributable to cardiovascular diseases (CVD). With an age-adjusted death rate of 272 per 100,000, the CVD-related mortality in India is much higher than the global average (235 per 100,000 population) [5]. The poor literacy rate with resultant lack of health awareness, inadequate public health system, and absence of national health insurance have resulted in poorer outcomes.

Out of all complications of CAD, ST-elevation myocardial infarction (STEMI) remains an absolute emergency. While early reperfusion has been shown to improve the outcomes, various registries have shown that the delay in presentation from the symptom onset has been an average of 300 minutes, far more than 140-170 minutes as reported in the Western world [6]. Due to delay in presentation, only 10% of STEMI patients undergo primary percutaneous coronary intervention (PCI) in India [6,7]. The various reasons quoted for delay are poor awareness and literacy rates, poor transport system, inadequate public health care system & insurance facilities, insufficient PCI-enabled centres, and in-hospital delays like unavailability of 24-hour catheterization laboratory, staff, and interventional cardiologist.

This study was conducted in a tertiary care centre to assess the various factors responsible for pre-hospital delay in acute coronary syndrome (ACS) patients.

## Materials and methods

It was an observational descriptive study conducted over a six-month period (April 2019 to September 2019) at a tertiary care centre in North India. All consecutive patients who were diagnosed to have ACS were included in the study after submitting informed consent. Patients not willing to participate in the study were excluded.

ACS was diagnosed as per the existing American College of Cardiology/American College of Cardiology/American Heart Association (ACC/AHA) and European Society of Cardiology (ESC) Guidelines [8-10]. STEMI was diagnosed as ST elevation of 1 mm or more in contiguous leads (except in V2/V3, where ST elevation of 2 mm and 1.5 mm is required in males and females respectively) along with clinical syndrome suggestive of ACS. Non ST-elevation myocardial infarction (NSTEMI) was diagnosed if a patient had chest pain/angina equivalents along with electrocardiogram (ECG) changes and significant rise in cardiac enzymes; whereas unstable angina (USA) was considered if a patient had these symptoms and ECG changes without any significant rise in cardiac enzymes. Non ST-elevation acute coronary syndrome (NSTE-ACS) included both NSTEMI and USA. A standardized tool was used for data collection which included interview schedule and consisted of socio-demographic data sheet, personal and clinical profile and questionnaire for symptom analysis, pathway followed for seeking medical treatment, factors related to pre-hospital delay and problems faced by patients to reach the hospital. The time of onset of symptoms, first appropriate medical contact, and time of presentation to this hospital were noted. The appropriate medical contact was defined as a centre that was capable of recognizing an ACS and starting guideline-directed medical treatment (GDMT) for ACS including thrombolysis for STEMI, dual antiplatelets, statins and anticoagulants. It may or may not be a PCI-enabled centre. The contact at any other medical centre was also noted. In addition, the mode of transport to the hospital was also noted.

Statistical analysis

The data was analysed by using Statistical Package for the Social Sciences version 23.0. (IBM Corp., Armonk, NY). Descriptive statistics (frequency, mean, median, range, standard deviation) were used and represented with help of tables and figures. Spearman’s rho correlation was utilized to study the correlation between different variables. P < 0.05 was considered significant.

## Results

**Figure 1:**
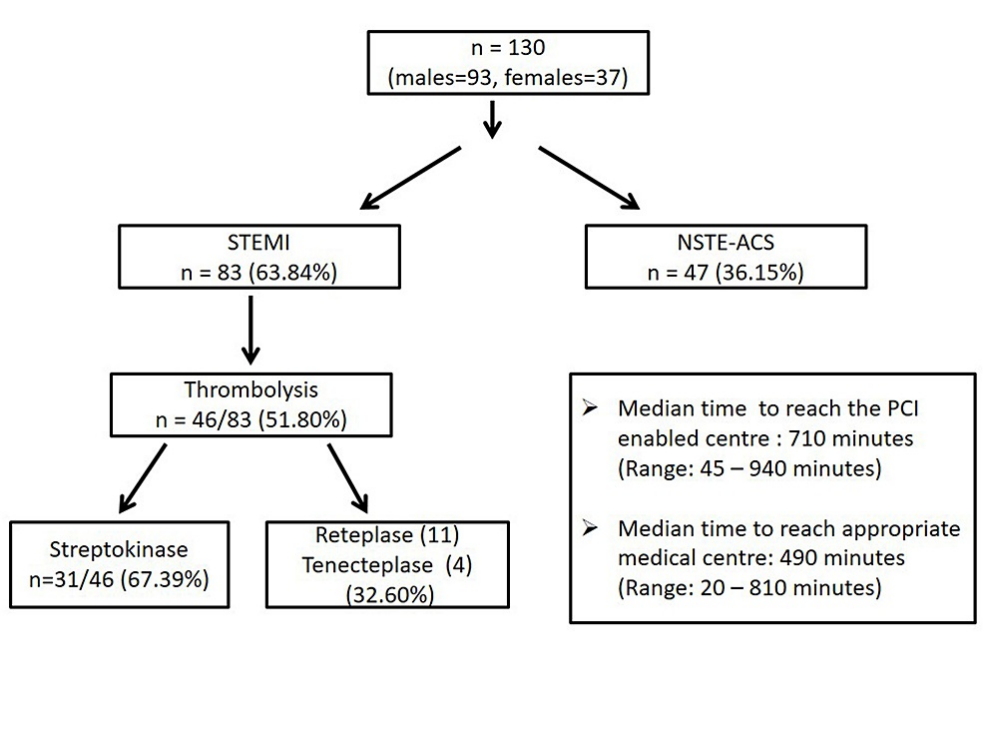
The cohort STEMI: ST-segment elevation myocardial infarction; NSTE-ACS: non ST-elevation acute coronary syndrome

A total of 130 ACS patients (males =93, females = 37) were studied. The mean age of patients was 61.3 ± 13.4 years. Eighty-three patients (63.84%) presented with STEMI; while 47/130 patients (36.15%) were diagnosed to have NSTE-ACS. The median time at which the ACS patients presented to the appropriate medical centre and PCI-enabled centre (this hospital) was 490 minutes (Range: 20 - 810 minutes) and 710 minutes (Range: 45 - 940 minutes). Out of all STEMI, 46/83 (51.80%) were thrombolysed. In the majority of patients (31/46, 67.39%), streptokinase was used for thrombolysis. In the rest of the patients, reteplase (11/46, 23.91%) and tenecteplase (4/46, 8.69%) were used. Figure 1 summarizes the cohort of our patients and their presentation. Coronary angiography and PCI in the same setting were done in 116/130 patients (89.23%, STEMI: 78/83, NSTE-ACS: 38/47) and 89/130 (68.46%, STEMI: 66/83, NSTE-ACS: 23/47) patients respectively. Five patients (STEMI, 2; NSTEMI, 3) died before an intervention could be carried out. The reasons for not undergoing intervention after coronary angiography were financial constraints (17/41: 41.14%), recanalized vessels (4/41, 9.75%) and triple vessel disease (6/41, 14.63%). Diabetes mellitus type 2 was present in 46/130 patients (35.38%), while hypertension was present in 50/130 (38.46%) patients. Eleven patients (8.46%) had previous history of PCI or coronary artery bypass graft surgery (CABG) and an additional six patients were under cardiology follow up for CAD. Out of 130 patients, 81 (62.30%) were from rural background. Table 1 summarizes the baseline characteristics of the enrolled patients.

**Table 1:** Baseline characteristics of the patients (n=130) STEMI: ST-segment elevation myocardial infarction; NSTE-ACS: non ST-elevation acute coronary syndrome; PCI: percutaneous coronary intervention

Characteristic	n=130	STEMI (n=83)	NSTE-ACS (n=47)
Mean age, years (± SD)	61.3 ± 13.4	56.4 ± 9.7	64.1 ± 10.2
Gender, n (%)	Male, 93 (71.5 %)	Males, 69 (83.1)	Males, 24 (51.)
	Female, 37 (28.5%)	Females, 14 (16.9)	Females, 23 (48.9)
Residence, n (%)	Urban, 49 (37.7%)	31 (37.3)	18 (38.3)
	Rural, 81 (62.3%)	52 (62.7)	29 (61.7)
Education status, n (%)	Literate, 93 (71.5%)	65 (78.3)	28 (59.6)
	Illiterate, 37 (28.5%)	18 (21.7)	19 (40.4)
Median annual per capita income, INR (Range)	42,900 (27,650 – 1,98,900)	44300 (26,154 – 1,87,980)	40930 (22,470 – 1,88,700)
Co-morbidities, n (%)	Hypertension, 50 (38.5%)	31 (37.3)	19 (40.4)
	Diabetes mellitus, 46 (35.4%)	25 (30.1)	21 (44.7)
	Coronary artery disease, 17 (13.1%)	6 (7.2)	11 (23.4)
	Cerebrovascular disease, 4 (3.1%)	2 (2.4)	2 (4.3)
	Chronic kidney disease, 6 (4.6%)	2 (2.4)	4 (8.5)
	Current smoking, 9 (6.9%)	5 (6.0)	4 (8.5)
Place of onset of symptoms, n (%)	Home, 105 (80.8)	70 (84.3)	35 (74.5)
	Work, 15 (11.5)	10 (12.0)	5 (10.6)
	Others, 10 (7.7)	3 (3.6)	7 (14.9)
Time of onset of symptoms, n (%)	Morning, 53 (40.8)	40 (48.2)	13 (27.7)
	Afternoon, 16 (12.3)	7 (8.4)	9 (19.1)
	Evening, 28 (21.5)	16 (19.3)	12 (25.5)
	Night, 33 (25.4)	20 (24.1)	13 (27.7)
Interpretation of ACS symptoms, n (%)	Yes, 42 (32.3)	28 (33.7)	14 (25.5)
	No, 88 (67.7)	55 (66.26)	33 (70.2)
Misinterpretation of cardiac symptoms with other symptoms, n (%)	Yes, 72 (55.4)	44 (53.01)	28 (59.57)
	No, 58 (44.6)	39 (47.0)	19 (40.4)
Perception of the symptoms to be serious, n (%)	Yes, 82 (63.1)	65 (78.3)	17 (36.2)
	No, 48 (36.9)	18 (21.7)	30 (63.8)
Knowledge or idea where to go for treatment, n (%)	Yes, 88 (67.7)	68 (81.9)	20 (42.6)
	No, 42 (32.3)	15 (18.07)	27 (57.4)
Availability of attendants during the onset of symptoms, n (%)	Yes, 116 (89.2)	76 (91.6)	40 (85.1)
	No, 14 (10.8)	7 (8.4)	7 (14.9)
Initial medical contact, n (%)	Local dispensary, 31 (23.8%)	5 (6.0)	26 (55.3)
	Appropriate medical centre (thrombolysis enabled), 84 (64.6%)	69 (83.1)	15 (31.9)
	PCI-enabled centre, 15 (11.5%)	9 (10.8)	6 (12.8)
Thrombolysis, n (%)	Initial medical contact	9 (10.8)	-
	PCI-enabled hospital	37 (44.6)	-
Distance to initial medical contact, n (%)	0-9 km, 86 (66.2)	59 (71.08)	27 (57.4)
	10-19 km, 33 (25.4)	19 (22.9)	14 (29.8)
	>20 km, 11 (8.4)	5 (6.02)	6 (12.7)
Conveyance, n (%)	Public transport, 12 (9.2%)	4 (4.8)	8 (17.0)
	Private transport, 113 (86.9%)	76 (90.6)	37 (78.7)
	Ambulance, 5 (3.9%)	3 (3.6)	2 (4.3)

**Figure 2:**
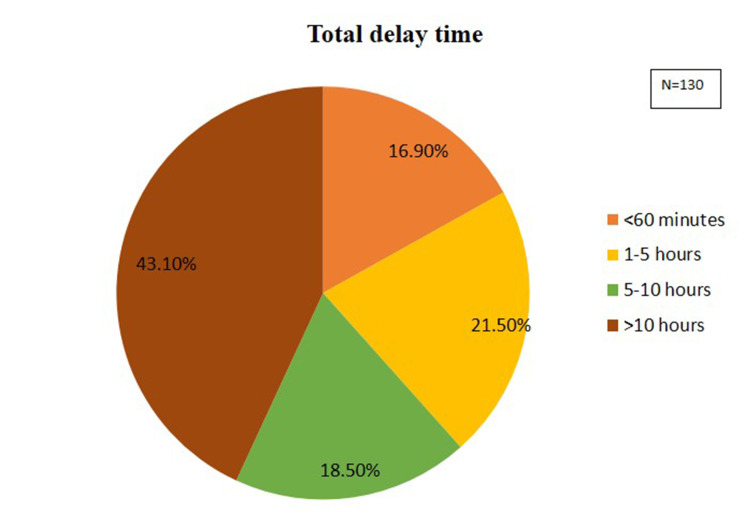
Total delay time in the study subjects.

The majority of the subjects, 105/130 (80.8%) were at home at the time of symptom onset and in 53/130 patients (40.8%), the onset of symptoms was in the morning hours. The most common symptom was chest discomfort (97/130, 74.61%). More than half of the patients (57.5%) misinterpreted the symptoms as non-cardiac and only 63.1 % considered them to be serious. Most of the patients (116/132, 89.2%) were accompanied by attendants at the time of onset of symptoms. Thirty-one patients (23.84%; all from rural backgrounds) reported to the local village dispensary after the onset of symptoms. These dispensaries were mostly manned by medical assistants. The most common medicines given to them at the dispensary were antacids (27/31, 87.09%), acetaminophen (22/31, 70.96%), ibuprofen (20/31, 64, 51%), and aspirin (20/31, 64.51%). None of them underwent an electrocardiogram (ECG) at the local dispensary. The median time wasted due to visit the local dispensary was 130 minutes (range: 40 - 380 minutes). None of the patients received GDMT at the local dispensary. Only five patients (3.9%) patients used the ambulance to reach the medical facility; all reached the PCI-enabled centre directly (mean time: 126 minutes ± 210 minutes). The median time wasted for arranging the conveyance was 110 minutes (range: 10 -210 minutes). Fifteen patients (11.6%) reached the PCI-enabled centre directly. In the majority of cases (66.2%), the distance from the place of symptom onset to the nearest medical centre (including the non - reperfusion-capable centre) was between 0-9 km. The majority of patients (43.1%) reached the appropriate medical centre after a delay of more than 10 hours. Figure 2 depicts the delay time in study subjects. Table 2 illustrates pre-hospital delay and socio-economic variables.

**Table 2:** Pre-hospital delay and socioeconomic variables PCI: percutaneous coronary intervention

	Mean time in reaching PCI-enabled centre (<300 minutes) n=56 (43.07%)	Mean time in reaching PCI-enabled centre (>300 minutes) n =74 (56.92%)	p-value
Gender, n (%)	Males, 41 (73.2)	Males, 52 (70.3)	0.714
	Females, 15 (26.8)	Females, 22(29.7)	
Residence, n (%)	Urban, 33 (58.9)	Urban, 16 (21.6)	< 0.0001
	Rural, 23 (41.1)	Rural, 58 (78.4)	
Visit to local dispensary, n (%)	Yes, 6 (10.7)	Yes, 25 (33.8)	0.0023
	No, 50 (89.3)	No, 49 (66.2)	
Education status, n (%)	Literate, 44 (78.6)	Literate, 49 (66.2)	0.1234
	Illiterate, 12 (21.4)	Illiterate, 25 (33.8)	
Median per capita income, INR (Range)	39,710 (27,650-1,76,500)	43,250 (33,400-1,98,900)	0.11
Median time to get means of conveyance, mins (Range)	90 (10-130)	150 (20-210)	0.03
Interpretation of ACS symptoms, n (%)	Yes, 29 (51.8)	Yes,13 (17.6)	<0.0001
	No, 27 (48.2)	No, 61 (82.4)	
Misinterpretation of cardiac symptoms with other symptoms, n (%)	Yes, 21 (37.5)	Yes, 51 (68.9)	0.0004
	No, 35 (62.5)	No, 23 (31.1)	
Perception of the symptoms to be serious, n (%)	Yes, 43 (76.8)	Yes, 39 (52.7)	0.005
	No, 13 (23.2)	No, 35 (47.3)	
Knowledge or idea where to go for treatment, n (%)	Yes, 47 (83.9)	Yes, 41 (55.4)	0.0006
	No, 9 (16.1)	No, 33 (44.6)	

The initial medical contact of STEMI patients (n=83) was a local dispensary in 5/83 patients (6.02%), thrombolysis-enabled centre in 69/83 patients (83.13%) and PCI-enabled hospital in 9/83 patients (10.84%). Despite being in window period and anticipated door to balloon time of more than 120 minutes due to logistic and financial reasons, thrombolysis was done only in nine patients (13.04%) and the rest were referred to PCI enabled hospital after ECG. All patients of STEMI were loaded with dual antiplatelets and high dose statins before transfer. The median time taken to reach the emergency of the PCI-enabled centre from the appropriate medical centre was 150 minutes (range 45-190 minutes). The reasons cited for transfer were anticipated door to balloon time <120 minutes (24/60, 40%), patients’ preference (10/60, 16.67%) and unknown reasons (26/60, 43.33%). In none of the circumstances, the catheterization laboratory or cardiac centre was informed in advance when the patient was transferred to a PCI-enabled centre. All patients reached the emergency and underwent pharmaco-invasive therapy.

The median per capita annual income of the cohort is Indian rupees (INR) 42,900/- (range: INR 27,650 - INR 1,98,900). A significant but weak negative correlation was found between per capita income, decision-making time and time taken to receive thrombolytic therapy. Likewise, a positive correlation between decision-making and the time taken to receive appropriate medical treatment was seen. Table 3 depicts the correlation between decision-making time and the time taken for thrombolytic therapy. Patients from rural backgrounds and the ones whose first medical contact was local dispensary were less likely to get thrombolysed and more likely to report late to PCI-enabled centres. The patients who had a previous history of CAD were more likely to report to the appropriate medical centre and to receive the appropriate medical treatment (p-value of 0.0003).

**Table 3:** Correlation of selected socio-demographic variables with decision-making time, time taken to receive thrombolytic therapy and appropriate medical treatment after onset of symptoms

Spearman's rho Correlation Coefficient		Time taken to receive thrombolytic therapy (n=46)	Decision-making time (n=130)	Time taken to receive appropriate medical treatment (n=130)
Age in years	Correlation coefficient value (P-value)	-0.080(0.574)	0.097(0.270)	-0.080(0.368)
Per capita income in Indian rupees (INR)	Correlation coefficient value (P-value)	-0.345(0.012)	-0.225(0.010)	-0.235(0.007)
Decision-making time (in minutes)	Correlation coefficient value (P-value)	0.522(0.001)	---	0.487(0.001)
Time taken to receive medical treatment (in hours)	Correlation coefficient value (P-value)	0.829(0.001)	0.487(0.001)s	---

## Discussion

Various Indian registries have documented a delay of 300-780 minutes after the onset of ACS symptoms [6,11-14]. Delay in GDMT in ACS patients leads to increased mortality and morbidity. This holds true especially for STEMI patients, where delay in presentation precludes the life-saving revascularization therapy and decreases the efficacy of thrombolytic therapy, even if the patients receive the same. The initial delay in presentation, lack of an adequate number of PCI-enabled centres, insufficient round the clock functional catheterization laboratories and financial constraints make primary PCI a distant dream in this part of the world. For these reasons, pharmaco-invasive therapy has been accepted as a close, though inferior alternative [6,11-14]. Even for pharmaco-invasive therapy, patients should present within 12 hours of symptom onset [8-10]. Beyond 12 hours, thrombolysis should be done only if the patient has ongoing chest pain or hemodynamic compromise; provided he/she cannot be taken up for coronary angiography [8-10]. In this study, we tried to evaluate the factors associated with the delay in the presentation of ACS patients to PCI-enabled centres.

The mean age (61.3 ± 13.4 years) and male preponderance (71.54%) in the index study were similar to the previous study done in the same centre [14]. STEMI was the most common presentation, similar to some other Indian studies [12,14]. The median time of presentation after the onset of symptoms to this hospital was 710 minutes; while the median time to reach an appropriate medical centre was 490 minutes which is also similar to as reported by other Indian studies [12,14]. Only around 50% of the STEMI patients could be thrombolysed, as documented earlier [11-14]. Similar to earlier studies, the main factors associated with the delay were non-recognition of cardiac symptoms (44.6%) and misinterpretation of symptoms as non-serious (36.9%) [15-18]. One-third of the patients could interpret the symptoms as ACS and only two-third of them had knowledge about the appropriate place for treatment.

The next important factor for the delay was the transport as documented in other studies as well [15,17]. Only five patients used the ambulance and the patients who reached the hospital within six hours of the onset of symptoms were able to get a conveyance at a shorter time interval. All patients who used ambulances arrived directly at the PCI-enabled centre. Similar to results from studies by Rajagopalan et al. [19] and Mohan et al. [20], the first medical contact at local dispensaries was associated with a significant delay in presentation. Beig et al. [17] Choudhary et al. [18] and Mohan et al. [20] reported rural residence as one of the factors associated with a significant pre-hospital delay which was also documented in our study.

Another important observation of the study was a failure to thrombolyse at the centre of first medical contact, even though it was thrombolysis-enabled centre. This delay in thrombolysis was due to the lack of judgement on the part of the referring clinician around 50% of the time. This highlights the need for enforcement of well-tested “hub-and-spoke” model for STEMI patients in the whole country [21]. Classifying the hospitals based on the availability of PCI and the distance from the PCI-enabled centre would produce uniformity in the management of STEMI patients, without any delay in following the appropriate revascularization therapy. Not only would it increase the rate of thrombolysis in STEMI patients as documented by Salve et al. (85% of patients were thrombolysed in this study), [22] but would also increase the rate of primary PCI by reducing the in-hospital delays. Similar to the observation made by Dracup [23] the patients with lower per capita income took more time for decision making and for thrombolysis.

As brought out by the present study and various other Indian studies as well, [17-22] to decrease the pre-hospital delay, public awareness about the ACS symptoms has to be increased. With the widespread use of the internet even in the remotest areas, mobile phones and advertisements on internet portals may be used to increase the awareness of the general public about the symptoms of ACS and the need for early reporting to appropriate medical centres. It can also be utilized to educate them about the appropriate medical centre to report to, in case they happen to develop ACS symptoms. Apart from improving the literacy rates, the health care system including the emergency care system, transport system and health insurance system needs major reforms, if we wish to provide the standard of care to our ACS patients. The well-tested “hub and spoke” systems need to interconnect the tertiary care centre with smaller hospital and primary care centres. Last but not the least, even the medical practitioners including doctors in peripheries need to reinforce the concepts of urgent diagnosis, early transfer, early thrombolysis and early transmission of information about STEMI patients to tertiary care centres.

Limitations

It is a single-centre study conducted at a tertiary care centre. The sample size is rather small. Since the study is based on questionnaires, the exact timing of onset of symptoms and reaching peripheral medical centre is based on patients’ and family’ responses to answers which may be affected by the recall bias.

## Conclusions

Our study concludes that socioeconomic status, rural residence, misinterpretation of symptoms, delay in getting transport and having local dispensary as first medical contact lead to a significant pre-hospital delay in ACS patients. Besides, judgemental errors on part of medical practitioners in the peripheries also significantly delay thrombolysis in STEMI patients. This calls for increased public awareness about ACS symptoms, improved health care and public transportation systems and continuous medical education of all medical practitioners to avoid pre-hospital delays of ACS patients.
